# Highly sensitive molecular diagnosis of prostate cancer using surplus material washed off from biopsy needles

**DOI:** 10.1038/bjc.2011.435

**Published:** 2011-10-18

**Authors:** R Bermudo, D Abia, A Mozos, E García-Cruz, A Alcaraz, Á R Ortiz, T M Thomson, P L Fernández

**Affiliations:** 1 Institut d’Investigacions Biomèdiques August Pi i Sunyer, c. Villarroel 170, 08036, Barcelona, Spain; 2 Department of Cell Biology, Institute for Molecular Biology, Science Research Council, c. Baldiri Reixac 15-21, 08028, Barcelona, Spain; 3 Bioinformatics Unit, Centro de Biología Molecular Severo Ochoa, Science Research Council and Universidad Autónoma de Madrid, c. Nicolás Cabrera 1, Cantoblanco, 28049, Madrid, Spain; 4 Department of Pathology, Hospital Clínic, c. Villarroel 170, 08036, Barcelona, Spain; 5 Department of Urology, Hospital Clínic, c. Villarroel 170, 08036, Barcelona, Spain; 6 School of Medicine, University of Barcelona, c. Casanova 143, 08036, Barcelona, Spain

**Keywords:** diagnosis, linear discriminant analysis, prostate cancer, real-time RT–PCR, gene signature, needle biopsy

## Abstract

**Introduction:**

Currently, final diagnosis of prostate cancer (PCa) is based on histopathological analysis of needle biopsies, but this process often bears uncertainties due to small sample size, tumour focality and pathologist's subjective assessment.

**Methods:**

Prostate cancer diagnostic signatures were generated by applying linear discriminant analysis to microarray and real-time RT–PCR (qRT–PCR) data from normal and tumoural prostate tissue samples. Additionally, after removal of biopsy tissues, material washed off from transrectal biopsy needles was used for molecular profiling and discriminant analysis.

**Results:**

Linear discriminant analysis applied to microarray data for a set of 318 genes differentially expressed between non-tumoural and tumoural prostate samples produced 26 gene signatures, which classified the 84 samples used with 100% accuracy. To identify signatures potentially useful for the diagnosis of prostate biopsies, surplus material washed off from routine biopsy needles from 53 patients was used to generate qRT–PCR data for a subset of 11 genes. This analysis identified a six-gene signature that correctly assigned the biopsies as benign or tumoural in 92.6% of the cases, with 88.8% sensitivity and 96.1% specificity.

**Conclusion:**

Surplus material from prostate needle biopsies can be used for minimal-size gene signature analysis for sensitive and accurate discrimination between non-tumoural and tumoural prostates, without interference with current diagnostic procedures. This approach could be a useful adjunct to current procedures in PCa diagnosis.

Early diagnosis of prostate cancer (PCa), the second most common cancer worldwide, is based on digital rectal examination and the determination of prostate-specific antigen (PSA) blood levels, followed by transrectal biopsy, an invasive procedure with potential side effects, mainly infections and haemorrhage (Ferlay *et al*, 2004). This combination of procedures is fraught with diagnostic uncertainties, such that ∼75% of biopsies prompted by an elevated PSA are subsequently found not to bear detectable PCa and around 15% of the patients with low PSA levels are subsequently found to harbour a tumour (Thompson *et al*, 2004; Schroder *et al*, 2009). In addition, the frequent focal involvement and small volume of tumours can hamper a definitive diagnosis. To increase diagnostic accuracy, an estimated 20% of biopsies are submitted to immunohistochemical detection of basal-cell markers and AMACR (*α*-methylacyl-CoA racemase). Still, up to 25% of these biopsies remain without a definitive diagnosis due to inconclusive results or tissue exhaustion (Browne *et al*, 2004; Montironi *et al*, 2006) and are classified as atypical small acinar proliferation (ASAP). Therefore, until more sensitive and specific procedures are developed, it is mandatory to extract the maximum information from such explorations.

The use of biological material that remains associated with aspiration or biopsy needles has been explored in different organs for morphological analysis, flow cytometry or molecular testing (Perry and Johnston, 1985; Stomper *et al*, 1998, 2001; Akimaru *et al*, 2004). In this study, we have explored the application of gene signatures on material washed off from biopsy needles in the diagnostic evaluation of prostate biopsies.

## Materials and methods

### Linear discriminant analysis

We previously identified a set of 318 genes whose expression levels are highly discriminant between malignant and normal prostate tissues (Bermudo *et al*, 2008). Microarray data sets from this study are deposited in the array express repository under accession number E-MEXP-1331. To the microarray data for the 27 samples of that study we added data for 57 unrelated samples, generated by Liu *et al* (2006). The resulting data set for all 84 samples (19 non-tumoural and 65 tumoural) was normalised with the robust multiarray average method and then quantile normalised (Irizarry *et al*, 2003, 2006). To identify minimal-size genesets capable of optimally discriminating between malignant and normal samples, we considered the expression values for the set of 318 genes in these 84 samples. We performed linear discriminant analysis (LDA) using three iterative approaches, two backward stepwise (a deterministic mode and a stochastic mode) and one forward stepwise. For the deterministic backward stepwise mode, we removed from the 318 geneset at each step the 10% of genes with the lowest contribution to the model and measured the classification accuracy at each step by leave-one-out-cross validation (LOOCV). For the stochastic backward stepwise mode, 50% of the genes from the group of the 20% least significant genes were randomly removed at each step, and the classification accuracy tested by LOOCV. We ran five trials in this way. For the forward stepwise LDA approach, data from all samples were used to generate 10 training-set and test-set pairs by randomly selecting 75% of the samples to form the training set, the remaining samples forming the test set. The best discriminant pair of genes from each possible pairwise combinations was initially selected from the training set as implemented in the MASS (Irizarry *et al*, 2006) package of R (Venables and Ripley, 2002), tested by LOOCV and used as a seed for increasing the number of genes, one at each round, keeping those that performed best as assessed by LOOCV. Genes with equal discriminating power were monitored and used to build all possible alternative models. The performance of the models generated in the training sets was measured by LOOCV, and those yielding a 100% classification success were applied to determine their classification accuracy on the corresponding test sets. The Fisher's linear discriminant function was calculated for each generated model.

For LDA of transcriptional data for 11 genes differentially expressed between non-tumoural and tumoural prostate tissues, we used data obtained from the real-time RT–PCR (qRT–PCR) analysis of samples washed off from prostate biopsy needles (see Materials and Methods). An LDA iterative forward stepwise mode was applied as described before to mean ΔCt data for these 11 genes in 28 tumoural and 26 non-tumoural samples.

### Processing of residual material recovered from simulated prostate biopsy needles

Freshly procured specimens were sampled with biopsy needles, which were inserted once in the peripheral zone of each lobe. The resulting tissue cores were removed for histopathological examination, the empty needle was washed in RNAlater (Ambion, Austin, TX, USA) and the recovered material stored until further processing. Thirty-seven samples thus obtained from 27 radical prostatectomies with adenocarcinoma were used as tumoural cases, in which the simulated biopsy cores contained tumoural glands. Eleven samples from tumour-free prostates were used as non-tumoural cases (Table 1). Total RNA was isolated using the RNeasy Kit (Qiagen, Valencia, CA, USA) and transcripts quantified by qRT–PCR (Table 2).

### Processing of prostate and colon tissues

Prostate tissues were obtained from patients undergoing radical prostatectomy for clinically localised prostate adenocarcinoma (14 non-tumoural and 34 tumoural; Table 3). Four normal colon mucosa samples were obtained from colectomy specimens from patients with colorectal adenocarcinoma. Total RNA was isolated as described before from tissue cryosections and used for qRT–PCR (Table 2). All tissue samples were obtained from the Tumour Bank-Biobank of the Hospital Clínic-IDIBAPS, Barcelona, and cleared for research use after local review board evaluation in compliance with current Spanish laws, including informed written consent.

### Processing of material washed off from transrectal prostate biopsy needles

Transrectal prostate biopsies were performed in patients with PSA levels >4 ng ml^−1^ with a 10-core scheme, using a single needle per patient. After each puncture, the tissue cylinder was removed and the empty needle was briefly washed in sterile saline solution in individual containers. All five tissue cores from one lobe were processed jointly for histopathology and given a single diagnosis. Washed-off samples were obtained from 27 patients diagnosed with adenocarcinoma in the biopsy cores and 26 patients with a non-tumoural diagnosis (Table 4). The latter had at least one additional benign prostate biopsy diagnosis, either previous or subsequent to the biopsy used in this study. For each patient, qRT–PCR analysis was performed on material washed off from one lobe, selecting for tumoural samples the lobe with the highest content of tumour, and for non-tumoural samples the lobe with representative benign histology. Washed-off samples were centrifuged, the pellet resuspended in RNA lysis buffer (Qiagen) and stored at −80 °C until use. Samples washed off from each needle puncture were processed separately to isolate total RNA followed by qRT–PCR (Table 2).

### Real-time RT–PCR

Total RNA was reverse-transcribed and cDNAs used for qRT–PCR on custom-designed TaqMan Low Density Arrays (Applied Biosystems, Foster City, CA, USA) in an ABI PRISM 7900HT instrument. Table 2 summarises probe information. Data were analysed using the SDS 2.3 software (Applied Biosystems). For simulated biopsies, relative transcript quantification was determined by the ΔΔCt method. For colonic and prostatic tissues, ΔCt data were used to build hierarchical clusters by UPGMA (unweighted pair group method with arithmetic mean) (Sneath and Sokal, 1973). Pvclust was used to calculate probability values for each cluster, using multiscale bootstrap resampling, with 10 000 simulations (Suzuki and Shimodaira, 2006). A cluster was considered as stable when the AU (approximately unbiased) *P*-values obtained were >95. For material washed off from transrectal prostate biopsy needles, individual washed-off samples with low input RNA (Ct (18S) >18.5) were discarded, cases with less than three washed-off samples with available valid qRT–PCR data were excluded from the analyses and mean ΔCt values of the available washed-off samples for each case were used for LDA.

## Results

### Generation of optimal gene signatures that discriminate non-tumoural from tumoural prostate tissues

With the aim of identifying minimal-size gene signatures that discriminate benign from tumoural prostate tissue, we applied LDA, which identifies a group of variables (here, gene expression levels) constituting a subset of a broader group, that can explain the data differences observed between two classes of entities (here, non-tumoural and tumoural samples). The linear function related to this signature can be used to assign new samples to its corresponding class. We have applied two backward stepwise and one forward stepwise approaches. The first two begin by building a model with all available genes and at each step remove a percentage of those that contribute less to the prediction of group membership, and the last builds up the model by testing all genes at each step and keeping the one that produces the most accurate classification for the next round.

**Figure 1:**
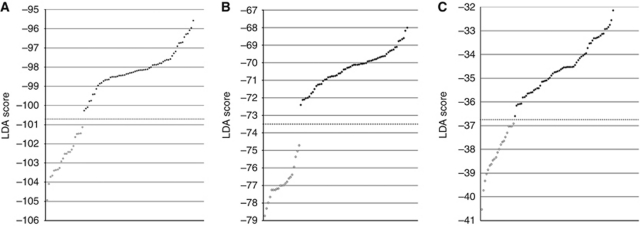
Linear discriminant analysis applied to microarray data for 318 genes discriminant between normal and tumoural prostate samples generates minimal-size gene signatures diagnostic of PCa. Results of the best models generated by each of the LDA approaches used: (**A**) backward stepwise deterministic, (**B**) backward stepwise stochastic and (**C**) forward stepwise. Plots show the performance of the models for the 65 tumoural (filled circles) and 19 normal (empty circles) samples analysed. LDA scores obtained by each sample are represented on the *y* axis. Samples are classified as tumoural or non-tumoural, when their corresponding LDA score are above or under the model threshold (dashed line), respectively. Note that each LDA model yields its own independent class-discriminant threshold.

For our LDA analyses, we considered as a starting point the 318 genes selected from a previous work (Bermudo *et al*, 2008) as those with the most significant differential expression between benign and adenocarcinoma prostate tissue samples. To the microarray data for the original 27 samples of that study we added data for 57 unrelated samples, generated by Liu *et al* (2006), resulting in a combined expression data set for a total of 84 samples, which included 19 non-tumoural and 65 tumoural samples. Linear discriminant analysis applied to the data for these 318 genes in all 84 samples produced 26 distinct signatures, all of which achieved 100% accuracy in classifying all 84 prostate samples as either benign or tumoural. Figure 1 shows the performance of the most interesting model obtained by each LDA approach, which corresponds to the one containing the smallest numbers of genes. With the deterministic backward stepwise approach, we obtained 17 models containing from 9 to 57 genes. Because this approach was initiated with a relatively high number of genes, 318, the contribution to sample classification of many genes in the first few iterative rounds was quite low, which could cause the loss of useful genes during the first rounds of iteration. Therefore, we have also applied a second approach consisting in the removal of genes, at each step, in a stochastic manner, from the group of less significant genes. In the backward stochastic approach, we obtained three models, containing from 18 to 23 genes. Supplementary Tables 1 and 2 show the genes contained in these models and the LDA loadings calculated for each gene.

Using the forward stepwise LDA approach, we generated and tested the models derived from all possible gene pairs independently for each of the 10 training sets. We obtained 49 gene pairs, ranked by their LOOCV classification accuracy, of which two pairs were repeated in three different training sets and five pairs in two training sets, while the remaining pairs were unique. The number of times that each gene appears in any of the selected pairs is summarised in Supplementary Table 3. Each of these pairs was used as a seed for its corresponding training set, and one gene was added at each step of the process. We collected the models generated (*n*=776) that achieved 100% classification accuracy as confirmed by LOOCV, and tested them on their corresponding test sets. We found that six models, each constituted by four genes, also classified perfectly (100% accuracy) those samples in three of the training-set/test-set groups (Supplementary Table 4).

These results show that different statistical approaches yield many minimal-size genesets that allow excellent discrimination between malignant and normal prostate tissues, based on their gene expression profiles.

### Classification of prostate biopsies as benign or tumoural by transcriptional profiling of surplus material from biopsy needles

Microarray hybridisation is not a first-choice technology in current clinical practice because of sample size requirements and cost issues. Practical diagnostic signatures would be those that can be used with alternative techniques, such as qRT–PCR. Given the significant differences in target sequences and intrinsic technological features, if microarray data are used as a starting point for these molecular diagnostic signatures, they must be first validated by qRT–PCR if this technique is to be applied for diagnostic purposes.

Because our discriminant model building based on 318 genes generated multiple optimal signatures, we next applied LDA by departing from a reduced subset of 11 genes that had been carefully validated by qRT–PCR (Bermudo *et al*, 2008), thus offering a significant gain in confidence in their discriminant capacity over genes not validated by this technique. Second, because our aim was to generate gene signatures with clinical applicability, while avoiding any interference with standard clinical practice, we decided to perform transcriptional profiling solely on the biological material that may remain attached to the biopsy needle, which is normally discarded after biopsy, leaving the biopsy cylinder entirely for routine diagnostic histopathological assessment.

**Figure 2:**
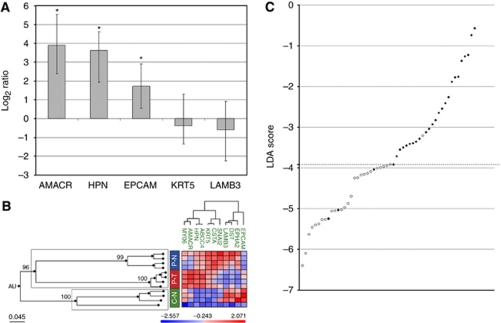
A highly sensitive and specific minimal-size gene signature diagnostic of PCa generated by LDA applied to qRT–PCR data for transrectal prostate biopsy needle wash-off samples. (**A**) Material washed off from simulated prostate biopsy needles is suitable for real-time RT–PCR transcript quantification. The bar plots represent the ratios of tumoural *vs* non-tumoural wash-off samples, showing the overexpression in carcinomatous washed-off samples of AMACR, HPN and EPCAM, as well as the underexpression of KRT5 and LAMB3. Non-tumoural washed-off samples were obtained from tumour-free prostates from nodular hyperplasias or radical cystoprostatectomies. Tumoural washed-off samples were obtained from radical prostatectomies diagnosed of adenocarcinoma and whose simulated biopsy cores contained tumoural glands. ^*^P-value <0.001. (**B**) Samples from colonic tissues display expression profiles for 11 selected genes that are distinct from normal and tumoural prostate samples. A hierarchical cluster was built with expression data obtained by qRT–PCR from non-tumoural colon (C–N) and tumoural (P–T) and non-tumoural (P–N) prostate samples. Pvclust analysis was used to assess the uncertainty of hierarchical clustering, obtaining AU values for each cluster, which show cluster stability when AU>95. Prostate data correspond to mean values from the available prostate samples. AU values are shown at the main branches of the hierarchical tree, illustrating that colonic samples display a gene expression profile that clearly differentiate them (AU>95) from all prostatic samples. (**C**) A highly sensitive and specific minimal-size gene signature diagnostic of PCa generated by LDA applied to qRT–PCR data for transrectal prostate biopsy needle wash-off samples. Results of the six-gene model generated by LDA, capable of discriminating wash-off samples from tumoural prostate biopsies (*n*=28) and non-tumoural ones (*n*=26). LDA scores obtained by each sample are represented in the *y* axis. Samples are classified as tumoural (filled circles) or non-tumoural (empty circles), when their corresponding LDA score is above or under the model's threshold (dashed line), respectively.

As a preliminary step to assess the feasibility of obtaining meaningful transcriptional profiles from such material, we simulated needle biopsies on prostates from radical prostatectomies, processed the tissue cores for pathological evaluation and washed the resulting empty biopsy needles to produce the material (needle ‘wash-offs’) for transcriptomic analysis. We confirmed that the amount and integrity of total RNA was adequate for qRT–PCR analysis (RNA integrity number higher than 5), and used these samples for qRT–PCR quantification of transcripts for five genes well known as differentially expressed in PCa: AMACR (Luo *et al*, 2002; Rubin *et al*, 2002), HPN (Dhanasekaran *et al*, 2001; Luo *et al*, 2001; Magee *et al*, 2001) and EPCAM (Strnad *et al*, 1989; Poczatek *et al*, 1999), overexpressed in PCa, and LAMB3 (Hao *et al*, 1996) and KRT5 (Ordonez, 1998; Abrahams *et al*, 2002), underexpressed in PCa. Our results show that three of these genes are also significantly differentially expressed in material washed off from simulated biopsy needles (Figure 2A), demonstrating the suitability of this material for sensitive transcript quantification.

In transrectal prostate biopsies, the needle traverses rectal tissues, is contaminated with peripheral blood and finally pierces the prostate capsule before reaching the prostatic glands and stroma. These tissues could potentially distort the profiles generated for the scarce biological material contained in needle washes and thus limit the discriminant power of gene signatures. Therefore, we analysed the expression of the 11-gene set in non-tumoural colorectal tissue, the major contaminant in actual prostate biopsies. The resulting profiles were clearly different (AU>95) from those for benign or tumoural prostate tissues (Figure 2B), suggesting that colorectal tissue that might attach to biopsy needles should not introduce a significant bias on prostate-specific transcriptional profiles.

We next assessed the applicability of gene signatures to residual material from actual prostate needle biopsies, for which we analysed material washed off from biopsy needles used on a total of 53 patients (Table 4). Transcript levels for the 11 genes were quantified by qRT–PCR and the resulting data used for LDA model building, in which the expression profiles were benchmarked against the histopathological diagnosis assigned to the corresponding biopsy tissues. The best discriminant model contained six genes (Table 5), four of which were overexpressed (ABCC4, AMACR, HPN and MYO6) and two underexpressed (CSTA and LAMB3) in PCa. By applying LDA with this gene signature, 25 of 28 samples with a tumoural histological diagnosis were assigned a tumoural molecular status, and 25 of 26 samples that lacked tumoural glands by histological evaluation were molecularly classified as non-neoplastic. Thus, our six-gene signature applied to biopsy needle residual material showed a 92.6% concordance with the pathological diagnosis of the corresponding biopsy cylinders, with a sensitivity of 88.8% and a specificity of 96.1% (Figure 2C).

Interestingly, nine of the biopsies whose corresponding washed-off samples were used required additional immunohistochemical analyses to reach a definitive histopathological diagnosis of PCa (six cases) or benign tissue (three cases). Eight out of these nine samples (88.8%) were correctly classified as tumoural or non-tumoural by the six-gene signature. These results suggest that transcriptional profiling of residual material recovered from prostate biopsy needles can achieve a diagnostic accuracy (tumoural *vs* non-tumoural) comparable to the combination of routine histological assessment and immunohistological analysis of biopsy cylinders.

## Discussion

Two important challenges in PCa diagnosis are the limitations of current serum markers for clinical screening and the limited sensitivity of biopsy techniques, both with a significant proportion of false or indeterminate results. The use of minimal genesets as molecular classifiers for tumour diagnosis or subclassification is therefore being actively explored in several neoplasms. We approached this problem by applying LDA to a set of 318 genes, which yielded multiple optimal signatures that discriminate non-tumoural from tumoural prostate tissues. The high probability of finding different discriminant solutions is rooted in the nature of transcriptomics, which considers numerous variables, thus increasing the likelihood of arriving at multiple discriminant models (Ein-Dor *et al*, 2005; Grate, 2005; Dougherty and Brun, 2007; Guillot *et al*, 2007). Many of these signatures contained non-overlapping genes, which were not necessarily those most differentially expressed between malignant and normal tissues. This is because genes with similar expression profiles across samples provide redundant information and are therefore discarded in the model building process. This could partially explain the divergences found between different studies describing diagnostic and prognostic signatures.

The main objective of this study was to maximise the diagnostic information obtained from prostate biopsies, for which we tested the applicability of gene signatures on surplus material obtained from biopsy needles that is normally discarded. The most discriminant model generated for such samples contained six genes, all previously associated with cancer, which confers our signature an additional functional relevance (Tomlins *et al*, 2007; Bermudo *et al*, 2008). This model showed 92.6% concordance between the molecular profiling of needle washes and the histopathological diagnosis of the corresponding biopsy cylinders. Therefore, our results indicate that molecular profiling performed on biological material that is routinely discarded, saline washes of biopsy needles, can achieve a diagnosis of prostate malignancy with an accuracy at least comparable to histopathological assessment of biopsy tissues. The fact that three cases were incorrectly classified as non-tumoural by molecular profiling might be explained by an insufficient representation of tumoural cells in that washed-off samples, given that the tissue from the biopsy and the washed-off sample are expected to be complementary but different samples. Due to ethical constraints, we have not performed molecular profiling on the biopsy cylinders. However, given the high sensitivity and specificity of our molecular diagnostic approach on residual material, it is reasonable to assume that profiling of the biopsy tissues themselves should afford even higher levels of diagnostic accuracy.

The high diagnostic accuracy of our approach is further highlighted by the fact that nine of the biopsies (16.6%) required immunohistochemical analysis in order to reach histopathological diagnosis, of which eight were initially diagnosed correctly by our molecular profiling approach. Of these cases, the only case initially classified as non-tumoural histologically but as tumoural by our approach might be found histologically positive for prostate adenocarcinoma in subsequent biopsies, which are not presently available.

Our six-gene signature was robust, discriminating benign from malignant samples independent of Gleason scores and tumour cell representations, which ranged from <5% to 90% of the total tissue. This suggests that it could be a useful adjunct for the management of biopsies with uncertain diagnoses. In Europe, there were 382 000 estimated new cases of PCa in 2008, implying that >1.5 million biopsies were performed, of which ∼300 000 could not be diagnosed by morphological criteria alone and required additional immunohistochemical assays (Ferlay *et al*, 2010). Importantly, even after immunohistochemical analysis, >70 000 cases were likely diagnosed as ASAP, which is associated with a PCa risk of around 40% in subsequent explorations (Montironi *et al*, 2006). Many of these diagnoses could potentially have been avoided with the application of complementary methods to enhance the sensitivity of routine biopsy diagnostic procedures.

We propose a hypothetical diagnostic course of action in which material washed off from prostate biopsy needles could be initially preserved, which implies a fast and economic way of collecting samples that are normally discarded with the biopsy needle. Those cases with a negative or uncertain histopathological diagnosis would be further processed for transcript quantification and analysis of our six-gene signature. Cases with a ‘molecular-negative’ result could resume a routine follow-up, whereas ‘molecular-positive’ cases would be advised immediate re-biopsy. Our method can be implemented without introducing additional surgical procedures or interfering with standard diagnostic procedures. Given the high sensitivity of our model on extremely scarce starting material, we further consider its possible use on prostate biological material sampled with fine-needle aspiration, whose smaller gauge would reduce side effects and patient discomfort associated to needle biopsies.

In conclusion, surplus biological material from prostate needle biopsies can be used for transcriptional profiling analysis to provide a useful adjunct to current diagnostic procedures, without causing any interference with the latter. Beyond the discriminant power afforded by our six-gene model to detect prostate tumours, our study highlights the many potential uses of a biological material so far neglected but potentially important for PCa management, including the accurate determination of multiple prognostic or predictive molecular markers or signatures. Finally, our approach could be also useful as an ancillary method in the clinical management of other types of neoplasia.

## Figures and Tables

**Table 1:** Clinical and histological characteristics of the patients whose samples were used for simulated prostate biopsies

**Tumoural samples**		**Normal samples** a	
Number of samples	37	Number of samples	11
Number of patients	27	Number of patients	6
Mean age (years)	62.32 (49–72)	Mean age (years)	68.5 (57–82)
Mean PSA (ng ml^−1^)	10.13 (3.09–55.8)	Mean PSA (ng ml^−1^)^b^	4.93 (2–7.1)
Median PSA (ng ml^−1^)	8	Median PSA (ng ml^−1^)^b^	5.3
Mean ratio free PSA/total PSA (%)^c^	15.3 (3.4–51.4)	Mean ratio free PSA/total PSA (%)^b^	23.47 (18.1–33.2)
Median ratio free PSA/total PSA (%)^c^	10	Median ratio free PSA/total PSA (%)^b^	21.3
			
*Gleason score (number of patients)*			
5	3		
6	3		
7	17		
8	2		
9	2		
			
*TNM stage (number of patients)*			
T2	15		
T3	12		
% of carcinoma in the tissue core^d^	38.4(<10–90)		

Abbreviation: PSA=prostate-specific antigen.

a Normal samples were obtained from tumour-free prostates from nodular hyperplasias and radical cystoprostatectomies.

b Data not available for two patients.

c Data not available for 10 patients.

d Mean content of tumoural glands relative to the whole tissue.

**Table 2:** Genes used in real-time RT–PCR analyses and their corresponding TaqMan probes

**Gene symbol**	**Description**	**Experiment** a	**Assay ID** b
ABCC4	ATP-binding cassette, subfamily C (CFTR/MRP), member 4	2, 3	Hs00195260_m1
AMACR	*α*-Methylacyl-CoA racemase	1, 2, 3	Hs00204885_m1
EPCAM	Epithelial cell adhesion molecule	1, 2, 3	Hs00158980_m1
HPN	Hepsin (transmembrane protease, serine 1)	1, 2, 3	Hs00170096_m1
MYO6	Myosin VI	2, 3	Hs00192265_m1
CSTA	Cystatin A (stefin A)	2, 3	Hs00193257_m1
DST	Dystonin	2, 3	Hs00794953_m1
EPHA2	EPH receptor A2	2, 3	Hs00171656_m1
KRT5	Keratin 5	1, 2, 3	Hs00361185_m1
LAMB3	Laminin, *β*3	1, 2, 3	Hs00165078_m1
SNAI2	Snail homologue 2 (Drosophila)	2, 3	Hs00161904_m1
RN18S1	RNA, 18S ribosomal 1	1, 2, 3	4342379-18S/Hs99999901_s1

Abbreviation: RT–PCR=reverse transcriptase polymerase chain reaction.

a Experiment in which each probe was used. 1: washed-off samples from simulated prostate biopsy needles; 2: whole tissue samples from prostate and colon; 3: washed-off samples from transrectal prostate biopsy needles.

b Manufacturer's probe ID.

**Table 3:** Clinical and histological characteristics of prostate whole tissue samples

**Tumoural samples**		**Normal samples**	
Number of patients	34	Number of patients	14
Mean age (years)	64.3 (50–74)	Mean age (years)	67 (56–73)
Mean PSA (ng ml^−1^)^a^	7.16 (3.58–19)	Mean PSA (ng ml^−1^)^a^	7.11 (4.6–10)
Median PSA (ng ml^−1^)^a^	6.8	Median PSA (ng ml^−1^)^a^	7.3
Mean ratio free PSA/total PSA (%)^b^	10.73 (6–23)	Mean ratio free PSA/total PSA (%)^c^	10.9 (7–15)
Median ratio free PSA/total PSA (%)^b^	9.6	Median ratio free PSA/total PSA (%)^c^	10.5
			
*Gleason score (number of patients)*			
5	6		
6	3		
7	21		
8	1		
9	3		
			
*TNM stage*			
T2	22		
T3	12		
Tumoural epithelium (%)^d^	60	Percentage of non-tumoural epithelium (%)^e^	43

Abbreviation: PSA=prostate-specific antigen.

a Data not available for one patient.

b Data not available for eight patients.

c Data not available for 14 patients.

d Mean content of tumoural glands relative to the whole tissue.

e Mean content of non-tumoural glands relative to the whole tissue.

**Table 4:** Clinical and histological characteristics of the patients whose prostate biopsy needles were used for residual sample retrieval and analysis

**Tumoural wash-off samples**		**Normal wash-off samples**	
Number of patients^a^	27	Number of patients	26
Mean age (years)	66 (52–84)	Mean age (years)	67 (57–78)
Mean PSA (ng ml^−1^)^b^	648 (1.47–10,230)	Mean PSA (ng ml^−1^)^c^	13.24 (2.11–79)
Median PSA (ng ml^−1^)^b^	8	Median PSA (ng ml^−1^)^c^	8.14
Mean ratio free PSA/total PSA (%)^d^	18.1 (6.9–34)	Mean ratio free PSA/total PSA (%)^e^	16.6 (9–24.7)
Median ratio free PSA/total PSA (%)^d^	15.9	Median ratio free PSA/total PSA (%)^e^	17.8
			
*Gleason score (number of patients)*			
6	10		
7	12		
8	0		
9	3		
10	2		
Mean % of carcinoma in the tissue core^f^	53.8 (<5–90)		

Abbreviation: PSA=prostate-specific antigen.

a For one patient, both lobes were analysed.

b Data not available for eight patients.

d Data not available for 15 patients.

c Data not available for six patients.

e Data not available for 10 patients.

f Mean content of tumoural glands relative to the whole tissue.

**Table 5:** Loadings of the six genes in the signature discriminant between non-tumoural and tumoural wash-off samples from transrectal prostate biopsies

**Gene**	**Loading**	**Description**
ABCC4	0.644969000296296	ATP-binding cassette, subfamily C (CFTR/MRP), member 4
AMACR	−0.336160831740741	*α*-Methylacyl-CoA racemase
CSTA	0.069085222	Cystatin A (stefin A)
HPN	−0.852895826962963	Hepsin (transmembrane protease, serine 1)
LAMB3	0.0403965255185185	Laminin, *β*3
MYO6	0.266395575314815	Myosin VI
