# Supplementary material for: Highly sensitive molecular diagnosis of prostate cancer using surplus material washed off from biopsy needles
Source: Br J Cancer. 2011 Oct 18;105(10):1600–7. doi: 10.1038/bjc.2011.435 (PMC3242534; doi:10.1038/bjc.2011.435)
Supplement: Supplementary Information [file bjc2011435x1.doc]

**Supplementary Table 1:** Genes contained in the best model obtained by the LDA backward stepwise deterministic approach, with their corresponding loadings in the discriminant function associated with the model.

| **Gene** | **Loading** | **Description** |
| --- | --- | --- |
| SLC8A1 | -4.09978 | solute carrier family 8 (sodium/calcium exchanger), member 1 |
| SLC25A12 | -3.00631 | solute carrier family 25 (mitochondrial carrier, Aralar), member 12 |
| VAMP3 | -2.77559 | vesicle-associated membrane protein 3 (cellubrevin) |
| ISYNA1 | -2.07460 | myo-inositol 1-phosphate synthase A1 |
| SNAI2 | -2.00033 | snail homolog 2 (Drosophila) |
| STAT5B | -1.69452 | signal transducer and activator of transcription 5B |
| FARSL | -1.51596 | phenylalanine-tRNA synthetase-like |
| HLF | 1.68985 | hepatic leukemia factor |
| NT5E | 2.15050 | 5'-nucleotidase, ecto (CD73) |

**Supplementary Table 2:** Genes contained in the best model obtained by the LDA backward stepwise stochastic approach, with their corresponding loadings in the discriminant function associated with the model.

| **Gene** | **Loading** | **Description** |
| --- | --- | --- |
| ILK | 4.12670 | integrin-linked kinase |
| GABRD | 3.30987 | gamma-aminobutyric acid (GABA) A receptor, delta |
| PDE2A | 2.96325 | phosphodiesterase 2A, cGMP-stimulated |
| DKC1 | 2.50854 | dyskeratosis congenita 1, dyskerin |
| RPL39 | 2.27285 | ribosomal protein L39 |
| CES1 | 1.72812 | carboxylesterase 1 (monocyte/macrophage serine esterase 1) |
| SFRS9 | 1.56232 | splicing factor, arginine/serine-rich 9 |
| PPP1R12B | -1.55736 | protein phosphatase 1, regulatory (inhibitor) subunit 12B |
| UMPK | -1.57551 | uridine monophosphate kinase |
| SNAI2 | -1.77891 | snail homolog 2 (Drosophila) |
| GNAZ | -2.91682 | guanine nucleotide binding protein (G protein), alpha z polypeptide |
| SLC25A12 | -2.99144 | solute carrier family 25 (mitochondrial carrier, Aralar), member 12 |
| ELF4 | -3.13898 | E74-like factor 4 (ets domain transcription factor) |
| CYB5R2 | -3.36494 | cytochrome b5 reductase b5R.2 |
| SLC8A1 | -3.57679 | solute carrier family 8 (sodium/calcium exchanger), member 1 |
| ISYNA1 | -4.59445 | myo-inositol 1-phosphate synthase A1 |
| EPB42 | -4.87978 | erythrocyte membrane protein band 4.2 |
| STK16 | -4.93029 | serine/threonine kinase 16 |

**Supplementary Table 3:** Genes contained in the 49 gene pairs generated by the LDA forward stepwise approach and the frequency with which they appear in these pairs.

| **Gene** | **Frequency** | **Description** |
| --- | --- | --- |
| KRT5 | 14 | keratin 5 |
| GPR161 | 7 | G-protein coupled receptor 161 |
| HPN | 6 | hepsin (transmembrane protease, serine 1) |
| ISYNA1 | 6 | myo-inositol 1-phosphate synthase A1 |
| FER1L3 | 5 | fer-1-like 3, myoferlin (C.elegans) |
| TP73L | 4 | tumor protein p73-like |
| FGFR2 | 4 | fibroblast growth factor receptor 2 |
| CAPG | 4 | capping protein (actin filament), gelsolin-like |
| LAMB3 | 4 | laminin, beta 3 |
| SHQ1 | 3 | SHQ1 homolog (S. cerevisiae) |
| COL17A1 | 2 | collagen, type XVII, alpha 1 |
| SNAI2 | 2 | snail homolog (Drosophila) |
| POLD2 | 2 | polymerase (DNA directed), delta 2, regulatory subunit 50kDa |
| SNX7 | 2 | sorting nexin 7 |
| EPCAM | 2 | epithelial cell adhesion molecule |
| SCRG1 | 2 | scrapie responsive protein 1 |
| ILK | 2 | integrin-linked kinase |
| GOLPH2 | 2 | golgi phosphoprotein |
| AKR1A1 | 1 | aldo-keto reductase family 1, member A1 (aldehyde reductase) |
| CLU | 1 | clusterin |
| ARHE | 1 | ras homolog gene family, member E |
| MPZL2 | 1 | myelin protein zero-like 2 |
| TSPAN13 | 1 | tetraspanin 13 |
| HSPB8 | 1 | heat shock 22kDa protein 8 |
| SND1 | 1 | staphylococcal nuclease and tudor domain containing 1 |
| MAOB | 1 | monoamine oxidase B |
| FOXA1 | 1 | forkhead box A1 |
| WDR23 | 1 | WD repeat domain 23 |
| CAV1 | 1 | caveolin 1, caveolae protein, 22kDa |
| TRIM29 | 1 | tripartite motif-containing 29 |
| LAPTM4B | 1 | lysosomal associated protein transmembrane 4 beta |
| GJB1 | 1 | gap junction protein, beta 1, 32kDa |
| ZMPSTE24 | 1 | zinc metalloproteinase (STE24 homolog, yeast) |
| PGLS | 1 | 6-phosphogluconolactonase |
| ABCC4 | 1 | ATP-binding cassette, sub-family C (CFTR/MRP), memb. 4 |
| EIF2AK1 | 1 | eukaryotic translation initiation factor 2-alpha kinase 1 |
| CLIC4 | 1 | chloride intracellular channel 4 |
| GBP2 | 1 | guanylate binding protein 2, interferon-inducible |
| KCNJ8 | 1 | potassium inwardly-rectifying channel, subfamily J, memb. 8 |
| KRT15 | 1 | keratin 15 |
| ATP2B4 | 1 | ATPase, Ca++ transporting, plasma membrane 4 |
| GSTP1 | 1 | glutathione S-transferase pi |
| NME1 | 1 | non-metastatic cells 1, protein (NM23A) expressed in |

**Supplementary Table 4:** The best discriminant models obtained by the LDA forward stepwise approach, with the associated Fisher’s discriminant function, the genes that form these signatures and their associated loadings.

| **Model** | **F*** | **Loading** | **Gene** | **Description** |
| --- | --- | --- | --- | --- |
| 1 - model 1 | 5.53 | 0.812 | MAPRE1 | microtubule-associated protein, RP/EB family, member 1 |
|  |  | -0.513 | FGFR2 | fibroblast growth factor receptor 2 |
|  |  | -1.749 | CLU | clusterin |
|  |  | -1.222 | PLEKHB1 | pleckstrin homology domain containing, family B (evectins) memb. 1 |
| 1 - model 2 | 5.76 | -0.762 | CAPG | capping protein (actin filament), gelsolin-like |
|  |  | -1.395 | FGFR2 | fibroblast growth factor receptor 2 |
|  |  | -1.502 | TCF7L1 | transcription factor 7-like (T-cell specific, HMG-box) |
|  |  | -1.034 | CORO1C | coronin, actin binding protein, 1C |
| 6 - model 1 | 6.73 | -1.544 | MAPRE1 | microtubule-associated protein, RP/EB family, member 1 |
|  |  | -1.790 | EPCAM | epithelial cell adhesion molecule |
|  |  | -1.882 | FGFR2 | fibroblast growth factor receptor 2 |
|  |  | 0.515 | ISYNA1 | myo-inositol 1-phosphate synthase A1 |
| 7 - model 1 | 6.59 | 0.600 | HPN | hepsin (transmembrane protease, serine 1) |
|  |  | -1.023 | LAMB3 | laminin, beta 3 |
|  |  | -1.563 | GPR161 | G-protein coupled receptor 161 |
|  |  | 0.897 | TRIB3 | tribbles homolog 3 (Drosophila) |
| 7 - model 2 | 6.62 | -1.302 | ATP6V1F | ATPase, H+ transporting, lysosomal 14kDa, V1 subunit F |
|  |  | -0.691 | HPN | hepsin (transmembrane protease, serine 1) |
|  |  | -1.606 | LAMB3 | laminin, beta 3 |
|  |  | 1.083 | GPR161 | G-protein coupled receptor 161 |
| 7 - model 3 | 6.46 | -0.877 | HPN | hepsin (transmembrane protease, serine 1) |
|  |  | -1.781 | PER2 | period homolog 2 (Drosophila) |
|  |  | 0.596 | LAMB3 | laminin, beta 3 |
|  |  | 1.279 | GPR161 | G-protein coupled receptor 161 |
| * Fisher's linear discriminant function | | | |  |
